# Mental health, eating disorder risk, and disordered eating patterns among Lebanese National Taekwondo Players: A cross-sectional study

**DOI:** 10.1371/journal.pone.0331975

**Published:** 2025-10-06

**Authors:** Maha Hoteit, Hassan Karaki, Amal Haidar, Rana Baroud, Habib Zarifeh, Ayoub Saidi, Fadi Kibbeh, Nathalie Jbeily, Zahra Sadek

**Affiliations:** 1 Faculty of Public Health, PHENOL Research program, Lebanese University, Hadath, Beirut, Lebanon; 2 Institut National de Santé Publique, d’Epidémiologie Clinique, et de Toxicologie (INSPECT-LB), Beirut, Lebanon; 3 Department of Primary Care and Population Health, University of Nicosia Medical School, Nicosia, Cyprus; 4 h Physical Therapy Department, Faculty of Public Health, Section I, Lebanese University, Beirut, Lebanon,; 5 Lebanese Taekwondo Federation, Sin el fil, Beirut, Lebanon; 6 Department of Physical Education and Sport Sciences, Faculty of Humanities and Social Sciences, University of Kurdistan, Sanandaj, Kurdistan, Iran; 7 Faculty of Education, University of Sciences and Arts in Lebanon, Beirut, Lebanon; 8 Faculty of Social and Political Sciences, University of Lausanne, Switzerland; 9 Physical Therapy Department, Faculty of Public Health, Islamic University of Lebanon, Khaldeh, Lebanon; Sheffield Hallam University, UNITED KINGDOM OF GREAT BRITAIN AND NORTHERN IRELAND

## Abstract

**Objectives:**

Elite athletes, particularly those engaged in combat sports like Taekwondo, are exposed to unique physical and psychological stressors. These demands may increase vulnerability to mental health challenges such as anxiety and depression, as well as disordered eating behaviors, especially when weight categories and performance pressures are involved. This study aimed to establish the mental health profile of Lebanese Taekwondo players while exploring the potential relationship between the psychological health indicators and eating disorders or disordered eating patterns.

**Methods:**

This cross-sectional study was conducted from January to July 2023 on 110 Lebanese black belt Taekwondo players recruited through the Lebanese Taekwondo Federation. Data collection involved anthropometric measurements, body composition analysis, and a self-administered questionnaire covering sociodemographic and socioeconomic factors, health behaviors, the **Sport Mental Health Assessment Tool-1 (SMHAT-1)** and the **Eating Attitudes Test-26 (EAT-26)**.

**Results:**

This study involved 110 Lebanese Taekwondo players (mean age 24.28 ± 10.5 years, 67.3% male), with the majority under 24 years old and half unemployed. Participants reported various socioeconomic challenges, including income reductions due to the post-economic crisis. Mental health assessments using the SMHAT-1 questionnaire revealed 14.5% were at high risk for anxiety, with female athletes exhibiting significantly higher anxiety scores (p = 0.006). Depression was reported in 13.6% of athletes, particularly among females (30% vs 5%, p = 0.000), and 10% had self-harm thoughts. Sleep disturbances affected 24.5% of participants, while 12.7% reported alcohol misuse. Disordered eating (DE) patterns were more common in females (50%) compared to males (p = 0.01), and the overall prevalence of DE was 33.6%. Using the EAT-26 questionnaire, 15.5% of participants were at risk of eating disorders (ED), with no significant gender differences. Factors such as unemployment, high exercise volume (≥10 hours per week), and reduced income were associated with higher ED risk. Depression and Psychosis were linked to both ED and DE, while anxiety was only associated with DE. Binary logistic models showed that athletes facing salary reductions were affected by ED, while those with psychosis had an 11-fold increased risk for ED. Household income and ADHD were strongly associated with DE patterns.

**Conclusion:**

This study revealed significant connections between mental health indicators and eating disorders or disordered eating patterns among Lebanese Taekwondo players. The findings highlight the need for targeted interventions, increased mental health awareness, and the development of prospective programs tailored to enhance athletes’ well-being and performance, including personalized mental health support.

## 1. Introduction

### 1.1. Perceptions and Psychological challenges of Taekwondo Athletes

Taekwondo athletes are often perceived as paragons of physical health and resilience [1]. The intense physical exertion, rigorous training routines, and the constant pursuit of excellence can lead to heightened stress, anxiety, and fatigue [2]. In addition, the demanding nature of their training and competition schedules, coupled with the pressure to perform at high levels, can expose them to a range of stressors that significantly impact their mental health [3]. Additionally, Taekwondo athletes face unique psychological challenges, including the fear of injury, the challenge of losing weight before competitions, performance anxiety, and the pressure to meet external expectations from coaches, fans, and sponsors [4]. These stressors can predispose these athletes to a variety of mental health issues, such as depression, anxiety disorders, and eating disorders [4]. Moreover, the competitive sports environment often emphasizes stoicism and mental toughness, which may discourage athletes from seeking help or expressing their psychological distress [2]. This can result in a lack of proper mental health support and interventions, exacerbating the risk of developing severe mental health problems and eating disorders [5].

### 1.2. Eating disorders and risk factors among elite Taekwondo athletes

Eating Disorders (EDs) are psychological and nutritional disorders that include the disturbance of eating behaviors and attitudes [6]. Risk factors such as dieting, weight loss prior to competitions, and body dissatisfaction have been considered predictors of EDs among Taekwondo athletes for many years [5]. A few examples of EDs are anorexia nervosa, bulimia nervosa, binge eating disorder, avoidant restrictive food intake disorder, and other specific feeding [7]. ED and disordered eating (DE) patterns are prevalent among elite athletes, with up to 19% of male athletes and 45% of female athletes suffering, and over 60% of female athletes experiencing body shaming pressure from coaches [8]. In general, EDs and DE patterns and psychotic disorders are linked among athletes [9]. However, the relationship between these three disorders remains unclear and under-studied [10]. For instance, Rodgers et al. (2022) found a significant relationship between diagnosed and probable attention deficit hyperactivity disorders (ADHD) and psychotic disorders, suggesting that non-athletic persons with these disorders are more likely to be diagnosed with an ED [10].

### 1.3. Contextual challenges for lebanese martial arts-Taekwondo athletes

It is important to discuss this topic among athletic people, especially those who are predisposed to ED and DE patterns. Despite the acknowledged importance of nutrition and psychology in sports, Lebanon currently lacks specific mental health and nutritional recommendations tailored to the unique needs of martial arts athletes and recurrent evaluation of their psychological status.

### 1.4. Study Aims and Research Objectives

The primary objective of this study was to establish profiles of the mental health of Lebanese Taekwondo players, the second objective is to examine the risk of ED and DE and investigate the potential relationship between these risks and psychological health.

## 2. Materials and methods

### 2.1. Sampe size calculation

The sample size of 110 athletes was deemed adequate based on methodological recommendations for logistic regression, requiring a minimum of 10 outcome events per predictor [11]. With up to eight predictors included in our models, the sample size allowed for stable estimates of associations between mental health and disordered eating indicators. Moreover, given the limited pool of eligible national-level Taekwondo black belt athletes in Lebanon, this sample reflects a considerable portion of the target population.

### 2.2. Study design and recruitment of study participants

This cross-sectional survey was conducted from January to July 2023 and involved 161 athletes. After applying eligibility criteria, 45 were excluded and 6 refused to fill in the questionnaire, leaving 110 eligible participants aligned with the study’s objectives. Inclusion criteria encompassed individuals aged 11–64 years, Lebanese nationals, holding a black belt in Taekwondo (including both coaches and players of both genders). Only black belt Taekwondo athletes were included in the study, as they represent individuals with higher levels of commitment, training intensity, and exposure to competitive pressures, which align with the study’s aim of examining psychological and behavioral risks in high-performance athletic settings. Exclusion criteria involved non-compliance with eligibility criteria and those who refused to fill in the questionnaire. The study used a convenient sampling method, reaching out to the Lebanese Taekwondo Federation and national team via email, involving a diverse group of athletes who provided consent, confidentiality, and eligibility screening before inclusion.

### 2.3. Data collection

The data collection process unfolded in two distinct phases over a 7-month period. Trained graduate dietitians, equipped with 3 days of training, conducted the assessments. The first phase initiated upon athlete notification. The study used standardized procedures to measure weight and height of participants.

#### 2.3.1. Questionnaire.

The self-administered questionnaire, composed of 4 main parts, was employed to collect the data between January and July 2023.The first part of the questionnaire assessed demographic and socioeconomic details, as well as medical history of study participants, including age, gender, governorate of residence, education level, marital status, employment status, role as a coach or player, involvement in professional sports, hours of workout per weekend, consumption of alcohol, tobacco, and energy drinks. The second part of the questionnaire was the Eating Attitudes Test (EAT-26) instrument, used to screen for eating disorders among our study participants [12]. EAT-26 includes sections A and B. Section A consists of 26 questions that are classified under the following three separate subscales: 1) ‘Dieting’, 2) ‘Bulimia and Food Preoccupation’, and 3) ‘Oral Control’. Each of the first 25 items in the EAT-26 is scored on a four- point scale: 0 (never, rarely, sometimes), 1 (often), 2 (usually) and 3 (always) and for the 26^th^ item is 26 is scored on an opposite four-point scale: 0 (always), usually, often), 1 (sometimes), 2(rarely) and 3 (never).The responses on the 26 items are summed at the end and a total score, ranging from 0 (minimum) to 78 (maximum), is extracted. A score of 20 or above was considered a cut-off point for identifying the possible presence of an eating disorder risk. Section B of the EAT-26 inspected the disordered eating behaviors of participants in the last 6 months (6 questions) [12]. The last part of the questionnaire was the Sport Mental Health Assessment Tool 1 (SMHAT-1) developed by the International Olympic Committee (IOC) is a standardized assessment tool designed to identify elite athletes who may be at risk for or already experiencing mental health symptoms and disorders [13]. Its primary goal is to enable the early identification of such issues, facilitating timely referral for appropriate support and treatment. The questionnaire comprises three steps, incorporating a total of 11 screening instruments. Participants begin by filling out Athlete Form 1, a triage tool for assessing mental health symptoms and disorders. The scoring determines whether the participant proceeds to Step 2 or discontinues the assessment. If the score falls between 10 and 16, no further action is needed. However, if the score ranges from 17 to 50, the athlete is directed to complete Athlete’s Form 2. Upon completion of Athlete’s Form 2, Step 2 is initiated, involving an assessment using six screening instruments for mental health symptoms and disorders. Based on the outcomes, participants proceed to Step 3a if all six screening instruments are under the threshold, and to Step 3b if one or more screening instruments are at or above the threshold. Step 3b involves a clinical assessment, supplemented with additional information gathered using Athlete’s Form 3, which includes screening instruments 7–11 [13].

### 2.4. Scoring of questionnaires

Specifically, the EAT-26 cutoff score of ≥20 was used to classify individuals at risk for ED, as recommended in the original validation studies. Similarly, categorical thresholds defined in the SMHAT-1 tool were applied for each mental health domain (e.g., GAD-7 ≥ 10 for moderate anxiety). In our analysis, all mental health and eating behavior scores were treated categorically (e.g., at-risk vs. not at-risk) for logistic regression models [12,13]. Statistical Analysis

Data analysis was performed using SPSS version 25.0. For the univariate analysis, data were presented as mean ± SD (Standard Deviation) for the continuous variables and as frequencies (N) and percentages (%) for the categorical ones. For bivariate analysis, the chi-squared test was used to examine associations between our categorical variables. For the chi-square test, when the minimum expected count was less than 5, Fisher’s exact test was used instead. The binary logistic regression analysis was applied to identify the determinants of EDs, and disordered eating. A p-value lower than 0.05 was considered significant.

### 2.5. Ethical considerations

The study protocol was approved by the Ethical Committee of the Lebanese University (#217/July 6, 2022), adhering to the Declaration of Helsinki principles. Participants provided informed consent, with a focus on confidentiality, privacy, and the voluntary nature of participation with the right to withdraw at any time. Written informed consent was obtained from the parents or legal guardians of all participating minors prior to data collection, in accordance with the requirements of the approved ethical protocol.

## 3. Results

### 3.1. Population characteristics

The demographic and socioeconomic characteristics of the study population are listed in Table 1. This study involved 110 participants with a mean age of 24.28 years (±10.5), predominantly male (67.3%). The majority were in the category age group < 24 years old (69.1%). Most participants were single (81.8%), and 60% had a university-level education. (Table 1.).

**Table 1:** Demographic, socioeconomic, training and lifestyle characteristics of overall study population and by sex.

		Overall (N%) (n = 110)			Male (N%) (n = 74)		Female (N%) (n = 36)		p-value
Age categories (years)	<24	76	69.1		46	62.6	30	83.4	0.067
	25- 60	34		30.9	28	37.8	6	16.70	
Marital Status	Married	19	17.3		14	18.9	5	13.9	0.618
	Single	90	81.8		59	79.7	31	86.1	
	Divorced	1	0.9		1	1.4	0	0	
Education status	University	66	60		44	59.5	22	61.1	0.656
	Secondary	32	9.1		23	31.1	9	25	
	Middle	11	10		6	8.1	5	13.9	
	Primary	1	0.9		1	1.4	0	0	
Current Occupation	Self-employed	16	14.5		12	16.2	4	11.1	0.150
	Part time job	18	16.4		11	14.9	7	19.4	
	Full time job	21	19.1		18	24.3	3	8.3	
	Not Working	55	50		33	44.6	22	61.1	
Governorate	Beirut and ML	84	76.3		54	73	30	83.3	0.125
	North	7	6.4		5	6.8	2	5.6	
	South	3	2.7		1	1.4	2	5.6	
	Beqaa and Hermel	16	14.5		14	18.9	3	5.6	
Household monthly income	Refused	21	19.1		16	21.6	5	13.9	0.209
	More than 500 USD	37	33.6		28	37.8	9	25	
	300-500 USD	20	18.2		11	14.9	9	25	
	0-300	32	29.1		19	25.7	13	36.2	
Reduction in monthly household income due to the economics crisis	No change	19	17.3		13	17.6	6	16.7	0.834
	Increase in salary	17	15.5		12	16.1	5	13.8	
	No more salary	38	34.5		23	31.1	15	41.7	
	No, half the salary	14	12.7		9	12.2	5	13.9	
	No, less than half the salary	22	20.0		17	23	5	13.9	
Type of training	Coach	25	22.7		22	29.7	3	8.3	0.017*
	Player	29	26.4		15	20.3	14	38.9	
	Both	56	50.9		37	50.0	19	52.8	
Workout (hours)	≤10	98	89.1		64	86.5	34	94.4	0.331
	>10	12	10.9		10	13.5	2	5.6	
Smoking Habit; cigarette or Hookah	Non-smoker	95	86.4		63	85.1	32	88.9	0.548
	Occasionally	8	7.3		5	6.8	3	8.3	
	Regularly	7	6.4		6	8.1	1	2.8	
Alcohol Consumption	No	66	60		46	62.2	31	86.1	0.198
	Occasionally	40	36.4		23	31.1	5	13.9	
	Regularly	4	3.6		5	6.8	0	0	
Energy-drink Consumption	No	78	70.9		47	63.5	31	86.1	0.036^*^
	Occasionally	27	24.5		22	29.7	5	13.9	
	Regularly	5	4.5		5	6.8	0	0	

* significant differences

Most of the (89.1%) participants reported spending <10 h/week exercising, while the remaining (10.9%) reported exercising more than 10 hours a week. Half of the participants were both players and coaches; the remaining were players or coaches, at 22.7% and 26.4%, respectively (Table 1). Of the taekwondo athletes, 86% did not smoke; nevertheless, 7.3% smoked occasionally and 6.4% frequently. Moreover, 60% and 71% drink neither alcohol nor energy drinks respectively, while occasional drinkers are 36.4% for alcohol and 24.5% for energy drinks and less than 10% are regular drinkers for both (Table 1).

### 3.2. Mental health scores of Taekwondo players using SMHAT-1 questionnaire

**Fig 1:**
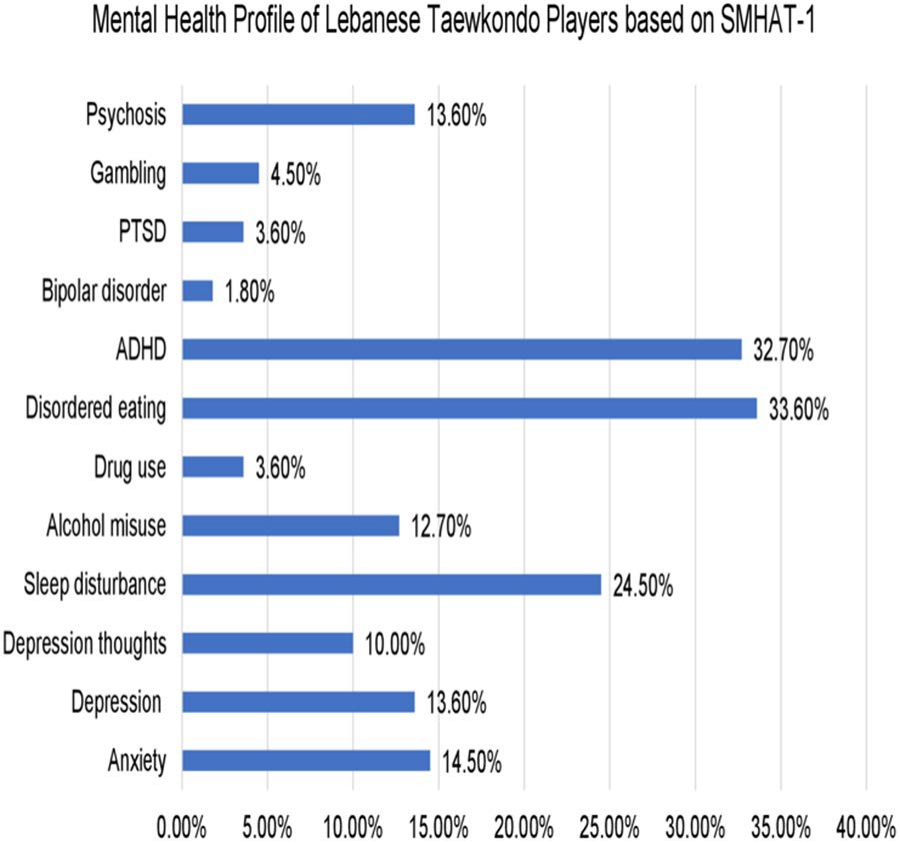
Mental health scores (SMHAT-1 QUESTIONNAIRE). *PTSD: post-traumatic stress disorder.

Using the SMHAT-1 questionnaire, 14.5% of Taekwondo athletes were identified as high risk for anxiety, with significantly higher scores among females compared to males (27.8% vs. 8%, p = 0.006). Depression was reported in 13.6% of athletes, again more prevalent among females (30% vs. 5%, p = 0.000), and 10% experienced thoughts of self-harm, with no gender differences observed. Sleep disturbances affected 24.5% of participants, and alcohol misuse was reported by 12.7%, both with no significant sex differences. Disordered eating (DE) patterns were more frequent in females (50%) than males (p = 0.01), with an overall prevalence of 33.6%. Additionally, 32.7% of athletes screened positive for ADHD, and 13.6% reported symptoms of psychosis. Other conditions—including drug use, gambling, bipolar disorder, and PTSD—were rare among the sample (S1 Table , Fig 1).

### 3.3. Eating disorders risk using EAT-26 questionnaire

**Fig 2:**
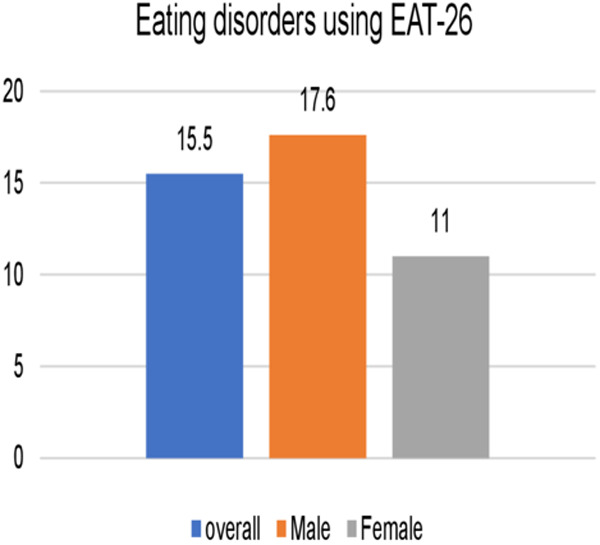
Eating disorders risk using EAT-26 questionnaire among Taekwondo players.

Using the EAT-26 questionnaire, the overall prevalence of ED risk was 15.5%; there was no significant difference in ED between males (17.6%) and females (11%) (p = 0.3) (Fig 2).

### 3.4. Correlations of different factors with ED and DE patterns among participants

Among participants who were unemployed, 23.6% are at high risk of ED compared to those who are employed (p = 0.01). Taekwondo players were more at risk to develop ED compared to coaches (p = 0.02). Not only that, participants who exercise 10 hours and more per week presented also high risk (p = 0.007). Sensitivity analyses by age group showed that the prevalence of disordered eating was higher among adolescents compared to adults, though patterns of association between mental health factors and ED risk were generally consistent across groups. No significant interaction effects between age group and primary outcomes were observed (p = 0.3). Around 9.5% of the participants who faced a reduction in monthly income were at risk of developing ED (p = 0.01). As for DE, females were at higher risk of developing DE patterns compared to males (p = 0.01). Those who are paid an income that is higher than 300$ were at high risk of having DE patterns compared to those who are paid lower (p = 0.002) (Table 2).

**Table 2:** The associations of demographic, socioeconomic, training and lifestyle characteristics of overall study population with ED and DE.

		ED_EAT26				p-value	DE_SMHAT-1				p-value
		No or low Risk		At Risk			No or low Risk		At Risk		
		N	N %	N	N %		N	N %	N	N %	
Sex	Male	61	82.4%	13	17.6%	0.3	55	74.3%	19	25.7%	0.01
	Female	32	88.9%	4	11.1%		18	50.0%	18	50.0%	
Age categories	<18 yrs	34	81.0%	8	19.0%	0.4	30	71.4%	12	28.6%	0.3
	>18 yrs	59	86.8%	9	13.2%		43	63.2%	25	36.8%	
Governorate	Beirut	69	82.1%	15	17.9%	0.5	59	70.2%	25	29.8%	0.3
	Mount Lebanon	6	85.7%	1	14.3%		3	42.9%	4	57.1%	
	South Lebanon	3	100.0%	0	0.0%		2	66.7%	1	33.3%	
	North Lebanon	15	93.8%	1	6.3%		9	56.3%	7	43.8%	
	Bekaa	0	0.0%	0	0.0%		0	0.0%	0	0.0%	
	Nabatiyeh	0	0.0%	0	0.0%		0	0.0%	0	0.0%	
	Baalbek-Hermel	0	0.0%	0	0.0%		0	0.0%	0	0.0%	
	Akkar	0	0.0%	0	0.0%		0	0.0%	0	0.0%	
Marital Status	Married	17	89.5%	2	10.5%	0.05	11	57.9%	8	42.1%	0.5
	Single	76	84.4%	14	15.6%		61	67.8%	29	32.2%	
	Divorced	0	0.0%	1	100.0%		1	100.0%	0	0.0%	
Education Status	University	59	89.4%	7	10.6%	0.1	40	60.6%	26	39.4%	0.2
	Secondary	23	71.9%	9	28.1%		22	68.8%	10	31.3%	
	Middle	10	90.9%	1	9.1%		10	90.9%	1	9.1%	
	Primary	1	100.0%	0	0.0%		1	100.0%	0	0.0%	
	Did not attend	0	0.0%	0	0.0%		0	0.0%	0	0.0%	
Current occupation	Unemployed	42	76.4%	13	23.6%	0.01	37	67.3%	18	32.7%	0.8
	Employed	51	92.7%	4	7.3%		36	65.5%	19	34.5%	
Type of Taekwondo players	Coach	22	88.0%	3	12.0%	0.02	19	76.0%	6	24.0%	0.2
	Player	20	69.0%	9	31.0%		21	72.4%	8	27.6%	
	Both	51	91.1%	5	8.9%		33	58.9%	23	41.1%	
Workout hours	<10h	73	90.1%	8	9.9%	0.007	51	63.0%	30	37.0%	0.2
	>10h	20	69.0%	9	31.0%		22	75.9%	7	24.1%	
Monthly income	<300$	47	88.7%	6	11.3%	0.2	43	81.1%	10	18.9%	0.002
	>300$	46	80.7%	11	19.3%		30	52.6%	27	47.4%	
Reduction in monthly income	No reduction	26	72.2%	10	27.8%	0.01	28	77.8%	8	22.2%	0.07
	Reduction	67	90.5%	7	9.5%		45	60.8%	29	39.2%	

Anxiety was not associated with eating disorders (ED) but was linked to disordered eating (DE) among participants (p = 0.000). Depression, however, was associated with both ED and DE (p = 0.03 and p = 0.000, respectively). Additionally, thoughts of self-harm related to depression were associated with DE (p = 0.02) but not with ED. Similarly, sleep disturbances, alcohol misuse, drug use, ADHD, PTSD, and gambling were associated with DE but not with ED, with gambling showing no significant association with any outcome (see Table 3). In contrast, psychosis was associated with both ED and DE (p = 0.005 and p = 0.000, respectively).

**Table 3:** The associations of the mental health profile of the participants with ED and DE.

		ED_EAT26				p-value	DE_SMHAT-1				p-value
		No or low Risk		At Risk			No or low Risk		At Risk		
		N	N %	N	N %		N	N %	N	N %	
Anxiety	Under threshold	81	86.2%	13	13.8%	0.2	70	74.5%	24	25.5%	0.000
	At or above threshold	12	75.0%	4	25.0%		3	18.8%	13	81.3%	
Depression	Under threshold	83	87.4%	12	12.6%	0.03	70	73.7%	25	26.3%	0.000
	At or above threshold	10	66.7%	5	33.3%		3	20.0%	12	80.0%	
Depression thoughts	Under threshold	84	84.8%	15	15.2%	0.7	69	69.7%	30	30.3%	0.02
	At or above threshold	9	81.8%	2	18.2%		4	36.4%	7	63.6%	
Sleep disturbance	Under threshold	72	86.7%	11	13.3%	0.2	64	77.1%	19	22.9%	0.000
	At or above threshold	21	77.8%	6	22.2%		9	33.3%	18	66.7%	
Alcohol misuse	Under threshold	81	84.4%	15	15.6%	0.8	68	70.8%	28	29.2%	0.009
	At or above threshold	12	85.7%	2	14.3%		5	35.7%	9	64.3%	
Drugs use	Under threshold	91	85.8%	15	14.2%	0.05	73	68.9%	33	31.1%	0.004
	At or above threshold	2	50.0%	2	50.0%		0	0.0%	4	100.0%	
DE	Under threshold	64	87.7%	9	12.3%	0.2	73	100.0%	0	0.0%	
	At or above threshold	29	78.4%	8	21.6%		0	0.0%	37	100.0%	
ADHD	Normal	63	85.1%	11	14.9%	0.8	64	86.5%	10	13.5%	0.000
	Symptoms highly consistent with ADHD	30	83.3%	6	16.7%		9	25.0%	27	75.0%	
Bipolar disorder	Normal	93	86.1%	15	13.9%	0.001	73	67.6%	35	32.4%	0.04
	Possible bipolar disorder	0	0.0%	2	100.0%		0	0.0%	2	100.0%	
PSD	sensitivity of 0.95 & specificity of 0.85	91	85.8%	15	14.2%	0.05	72	67.9%	34	32.1%	0.07
	sensitivity of 0.83 & specificity of 0.91	2	50.0%	2	50.0%		1	25.0%	3	75.0%	
Gambling	Non problem gambling	88	83.8%	17	16.2%	0.32	71	67.6%	34	32.4%	0.2
	Problem gambling with negative consequences and a possible loss of control	5	100.0%	0	0.0%		2	40.0%	3	60.0%	
Psychosis	normal	84	88.4%	11	11.6%	0.005	70	73.7%	25	26.3%	0.000
	At risk of psychosis	9	60.0%	6	40.0%		3	20.0%	12	80.0%	

### 3.5. Determinants of ED and DE in the studied population

In the analysis of determinants of ED and DE, binary logistic regression was executed. The study investigated factors influencing eating disorder risk and disordered eating patterns. The resulting models revealed significant predictors (Tables 4 and 5).

**Table 4:** Determinants of eating disorder risk using EAT-26.

	B	S.E.	Wald	df	Sig.	Exp(B)	95% C.I.for EXP(B)	
							Lower	Upper
Psychosis	2.470	1.093	5.108	1	.024	11.821	1.388	100.655
Reduction in monthly incomes	−2.200	.791	7.740	1	.005	.111	.024	.522

* Significant at p-value < 0.05

**Table 5:** Determinants of disordered eating patterns using SMHAT-1.

Variables	B	S.E.	Wald	df	Sig.	Exp(B)	95% C.I.for EXP(B)	
							Lower	Upper
Monthly income >300$ vs < 300$	2.711	.873	9.632	1	.002	15.043	2.715	83.339
ADHD (At risk vs No or low risk)	3.369	.867	15.103	1	.000	29.054	5.312	158.902

Using binary logistic models, it was shown that people who faced reduction in their salary due to the economic crisis are at risk of having ED compared to those who didn’t find any challenges regarding reduction of monthly salary (OR=0.11; 95% CI 0.02–0.5]. In addition, those who are at high risk of having psychosis were having 11 times higher risk to have ED compared to normal people (OR=11.8; 95% CI [1.3–100]) (Table 4).

As for DE, household monthly income (>300$) and ADHD were strongly associated with DE patterns ([OR=15 [2.7–83] and OR=29 [5–158]) respectively) (Table 5).

## 4. Discussion

This study aimed to explore the association between various socioeconomic, psychological, and behavioral factors with eating disorders (ED) and disordered eating (DE) patterns among Lebanese Taekwondo athletes. The findings revealed substantial psychological challenges among participants, with high rates of anxiety (14.5%), depression (13.6%), and self-harm thoughts (10%), particularly among females. ADHD and psychosis were also prevalent (32.7% and 13.6%, respectively), along with sleep disturbances (24.5%). The overall prevalence of ED was 15.5%, with no significant gender difference, while DE was reported by 33.6% of athletes, more commonly in females (50%). These findings underscore the need for targeted mental health and nutritional support in this vulnerable population.

### 4.1. Socioeconomic and behavioral factors associated with ED and DE

Unemployment and income reduction emerged as significant predictors of ED risk. Unemployed athletes had higher ED risk (23.6%, *p* = 0.01), and those reporting a decrease in monthly income also showed increased vulnerability (*p* = 0.01). These results align with literature linking financial instability to disordered eating through increased stress and reduced access to balanced nutrition [14,15]. Moreover, high physical activity (>10 hours/week) was significantly associated with ED risk (*p* = 0.007), likely due to weight management pressures inherent in combat sports like Taekwondo [15–16].

For DE, females were found to be at a higher risk compared to males (*p* = 0.01), which is consistent with broader studies on gender differences in disordered eating, where women in weight-sensitive sports may face heightened pressures to maintain a lean physique [17]. Interestingly, participants earning more than $300 per month were also at greater risk of developing DE (*p* = 0.002). While this may appear counterintuitive, several studies have reported that higher socioeconomic status can be associated with increased exposure to societal ideals of thinness and body image dissatisfaction [17,18]. Among athletes, greater financial resources may enable access to strict dietary regimens, supplements, or body-enhancing strategies—factors that could inadvertently reinforce DE behaviors [19,20]. Additionally, athletes with higher income may experience greater pressure to perform and conform to specific aesthetic or weight standards [21]. These dynamics suggest that in certain athletic subgroups, socioeconomic advantage might not be protective against DE, but rather a contributing factor through elevated expectations and access to appearance-related modifications.

Given the broad age range of participants, we conducted stratified analyses to explore potential developmental differences in ED and mental health risks. The higher prevalence of disordered eating observed in adolescent athletes aligns with previous literature emphasizing vulnerability during adolescence [20]. However, the consistency of associations across age groups suggests that the identified risk factors may operate similarly throughout different developmental stages [21–22]. Nevertheless, future research with larger samples in specific age groups is warranted to further elucidate age-specific mechanisms and tailor interventions accordingly.

### 4.2. Mental health conditions and disordered eating

Mental health factors were strongly associated with DE patterns. Anxiety and depression were both significantly linked to DE (*p* = 0.000), with depression also associated with ED (*p* = 0.03). Self-harm thoughts were specifically related to DE (*p* = 0.02), reflecting deeper psychological distress in this group. Several other mental health conditions, sleep disturbances, alcohol misuse, drug use, ADHD, and PTSD, were also associated with DE but not ED, indicating that DE may serve as a broader coping mechanism for emotional and behavioral dysregulation in athletes [23–26].

ADHD showed the strongest association with DE (OR = 29; 95% CI [5–158]), suggesting that impulsivity and poor emotional control could predispose athletes to irregular eating behaviors [26].

### 4.3. Psychosis and its relationship with ED and DE

Psychosis was significantly associated with both ED and DE (*p* = 0.005 and *p* = 0.000, respectively). Athletes at high risk of psychosis had an 11-fold increased risk of ED (OR = 11.8; 95% CI [1.3–100]), reinforcing the link between severe mental illness and disordered eating, potentially driven by distorted body perception or maladaptive coping strategies [27].

### 4.4. Multivariate insights

Logistic regression analyses further clarified key associations. The binary logistic regression models further clarified the strength of associations between various factors and ED or DE. Interestingly, participants who experienced a reduction in salary due to the economic crisis had a significantly lower likelihood of ED (OR = 0.11; 95% CI [0.02–0.5]). Although this finding appears paradoxical and contrasts with established evidence linking financial hardship to increased risk of disordered eating, it may reflect context-specific coping mechanisms within this athletic population. For example, individuals facing economic strain may reduce their engagement with resource-intensive or appearance-focused behaviors, such as specialized diets or supplements. Alternatively, the result may indicate a shift in priorities away from body image concerns during financial hardship. This unexpected association warrants cautious interpretation and further investigation in future studies [28,29].

## 5. Limitations and strengths

This study’s cross-sectional design prevents determining causality. Additionally, self-reported data may introduce bias, with some responses possibly underreported or overreported. Although the EAT-26 and SMHAT-1 questionnaires are validated tools, their length (20-40 minutes to complete) made them impractical, potentially affecting participants’ motivation and response accuracy. This likely contributed to the low participation rate, with only 110 out of 161 athletes completing the questionnaire. The lack of clinical diagnoses for high-risk participants limits the ability to confirm the accuracy of the results. Furthermore, the convenience sampling method may limit the generalizability of the findings. The exclusive focus on black belt athletes may limit the generalizability of the findings to lower-level or recreational Taekwondo practitioners, who may experience different levels of stress, competition, and training-related pressures Despite these limitations, this study, the first of its type in Lebanon and the Middle East region, provides valuable insights into the mental health and eating disorder risks among Lebanese Taekwondo players, calling for further research to address these issues and improve the validity of future findings.

## 6. Conclusion

In conclusion, this study highlights the complex interplay of psychological, socioeconomic, and sport-related factors contributing to the risk of eating disorders (ED) and disordered eating (DE) among Taekwondo athletes. Mental health challenges such as depression, anxiety, and ADHD emerged as significant predictors of disordered eating behaviors. Additionally, unemployment and fluctuations in income were found to influence the risk of ED and DE, reflecting the broader impact of economic stress on athlete well-being. The association between excessive physical activity and ED, particularly in a weight-sensitive sport like Taekwondo, underscores the importance of monitoring training intensity and body image pressures. Given the unique performance demands placed on these athletes, developing tailored interventions that integrate both psychological support and nutritional care is essential to prevent and manage ED and DE in this vulnerable population.

## Supporting information

S1 Table Mental health scores based on SMHAT-1 questionnaire. (DOCX)
