# Supplementary material for: Mental health, eating disorder risk, and disordered eating patterns among Lebanese National Taekwondo Players: A cross-sectional study
Source: PLoS One. 2025 Oct 6;20(10):e0331975. doi: 10.1371/journal.pone.0331975 (PMC12500144; doi:10.1371/journal.pone.0331975)
Supplement: S1 Table — (DOCX) [file pone.0331975.s001.docx]

|  | | **Overall** | | **Male** | | **Female** | |  |
| --- | --- | --- | --- | --- | --- | --- | --- | --- |
|  |  | N | N% | N | N % | N | N % | p-value |
| Anxiety | Under threshold | 94 | 85.5% | 68 | 91.9% | 26 | 72.2% | 0.006 |
|  | At or above threshold | 16 | 14.5% | 6 | 8.1% | 10 | 27.8% |  |
| Depression | Under threshold | 95 | 86.4% | 70 | 94.6% | 25 | 69.4% | 0.000 |
|  | At or above threshold | 15 | 13.6% | 4 | 5.4% | 11 | 30.6% |  |
| Depression thoughts | Under threshold | 99 | 90.0% | 69 | 93.2% | 30 | 83.3% | 0.10 |
|  | At or above threshold | 11 | 10.0% | 5 | 6.8% | 6 | 16.7% |  |
| Sleep disturbance | Under threshold | 83 | 75.5% | 58 | 78.4% | 25 | 69.4% | 0.3 |
|  | At or above threshold | 27 | 24.5% | 16 | 21.6% | 11 | 30.6% |  |
| Alcohol misuse | Under threshold | 96 | 87.3% | 65 | 87.8% | 31 | 86.1% | 0.7 |
|  | At or above threshold | 14 | 12.7% | 9 | 12.2% | 5 | 13.9% |  |
| Drugs use | Under threshold | 106 | 96.4% | 72 | 97.3% | 34 | 94.4% | 0.4 |
|  | At or above threshold | 4 | 3.6% | 2 | 2.7% | 2 | 5.6% |  |
| Disordered eating | Under threshold | 73 | 66.4% | 55 | 74.3% | 18 | 50.0% | 0.01 |
|  | At or above threshold | 37 | 33.6% | 19 | 25.7% | 18 | 50.0% |  |
| ADHD | Normal | 74 | 67.3% | 53 | 71.6% | 21 | 58.3% | 0.16 |
|  | Symptoms Highly Consistent with ADHD | 36 | 32.7% | 21 | 28.4% | 15 | 41.7% |  |
| Bipolar disorder | Normal | 108 | 98.2% | 72 | 97.3% | 36 | 100.0% | 0.32 |
|  | Possible bipolar disorder | 2 | 1.8% | 2 | 2.7% | 0 | 0.0% |  |
| PTSD | sensitivity of 0.95 & specificity of 0.85 | 106 | 96.4% | 72 | 97.3% | 34 | 94.4% | 0.45 |
|  | sensitivity of 0.83 & specificity of 0.91 | 4 | 3.6% | 2 | 2.7% | 2 | 5.6% |  |
| Gambling | Non problem gambling | 105 | 95.5% | 71 | 95.9% | 34 | 94.4% | 0.7 |
|  | Problem gambling with negative consequences and a possible loss of control | 5 | 4.5% | 3 | 4.1% | 2 | 5.6% |  |
| Psychosis | Normal | 95 | 86.4% | 68 | 91.9% | 27 | 75.0% | 0.01 |
|  | At risk of psychosis | 15 | 13.6% | 6 | 8.1% | 9 | 25.0% |  |
